# A systematic review of zoonotic enteric parasitic diseases among nomadic and pastoral people

**DOI:** 10.1371/journal.pone.0188809

**Published:** 2017-11-30

**Authors:** Amber N. Barnes, Anu Davaasuren, Uyanga Baasandagva, Gregory C. Gray

**Affiliations:** 1 Institute of Veterinary Medicine, Ulaanbaatar, Mongolia; 2 Division of Infectious Diseases, School of Medicine, Duke University, Durham, NC, United States of America; 3 National Center for Communicable Disease, Ulaanbaatar, Mongolia; 4 National Center for Zoonotic Disease, Ulaanbaatar, Mongolia; 5 Global Health Institute, Duke University, Durham, NC, United States of America; Michigan State University College of Veterinary Medicine, UNITED STATES

## Abstract

**Introduction:**

Zoonotic enteric parasites are ubiquitous and remain a public health threat to humans due to our close relationship with domestic animals and wildlife, inadequate water, sanitation, and hygiene practices and diet. While most communities are now sedentary, nomadic and pastoral populations still exist and experience unique exposure risks for acquiring zoonotic enteric parasites. Through this systematic review we sought to summarize published research regarding pathogens present in nomadic populations and to identify the risk factors for their infection.

**Methods:**

Using systematic review guidelines set forth by PRISMA, research articles were identified, screened and summarized based on exclusion criteria for the documented presence of zoonotic enteric parasites within nomadic or pastoral human populations. A total of 54 articles published between 1956 and 2016 were reviewed to determine the pathogens and exposure risks associated with the global transhumance lifestyle.

**Results:**

The included articles reported more than twenty different zoonotic enteric parasite species and illustrated several risk factors for nomadic and pastoralist populations to acquire infection including; a) animal contact, b) food preparation and diet, and c) household characteristics. The most common parasite studied was *Echinococcosis* spp. and contact with dogs was recognized as a leading risk factor for zoonotic enteric parasites followed by contact with livestock and/or wildlife, water, sanitation, and hygiene barriers, home slaughter of animals, environmental water exposures, household member age and sex, and consumption of unwashed produce or raw, unprocessed, or undercooked milk or meat.

**Conclusion:**

Nomadic and pastoral communities are at risk of infection with a variety of zoonotic enteric parasites due to their living environment, cultural and dietary traditions, and close relationship to animals. Global health efforts aimed at reducing the transmission of these animal-to-human pathogens must incorporate a One Health approach to support water, sanitation, and hygiene development, provide education on safe food handling and preparation, and improve the health of domestic animals associated with these groups, particularly dogs.

## Introduction

As long as life has existed on earth, there have been parasites [1]. In fact, there is not a single organism that is protected against parasites [1]. Humans have been hosts to parasites across antiquity and the study of this relationship among early civilizations lead to the creation of the field of paleoparasitology [2]. Paleoparasitologists are gaining insight into which parasite species may have co-evolved with humans and which ones were initially found in localized environments, then spread as humans migrated across the globe and began using new technologies, instituted innovative agricultural practices, lived in more urbanized settings, and domesticated animals [1,3–5]. This discipline compliments the One Health approach of inclusive and collaborative research efforts across expert fields to increase the health and well being of humans, animals and the environment and provides insight into the current human-animal-parasite relationships of today [6].

Due to the cultural and behavioral changes of humans, the parasitic landscape of the world has been altered and new host systems have been created and novel environments infiltrated [3–4]. In particular, humans have been exposed to an increasing number of zoonotic foodborne parasites throughout our species history due to the close association between humans and domestic animals, encroachment into landscapes previously reserved for wildlife, climate change resulting in modified flora and fauna, revolutions in cooking methods, diet and food availability, and in vogue culinary items expanding throughout societies [1,3,5,7]. These gastrointestinal pathogens are found worldwide and can lead to diarrhea, malnutrition, problems with the central nervous system/neurological disorders, epilepsy, reproductive and congenital disorders, cancer, and even death [8]. And despite global advances in food safety standards, humans remain at risk for exposure to food and waterborne illness, including parasitic zoonoses [9].

Zoonotic enteric parasites (ZEP) use animals and humans as hosts and are typically transmitted through ingestion of contaminated food, water, soil, or fomites [10]. ZEPs of public health concern for humans span three taxonomic kingdoms: Animalia, including helminths of cestodes (ex. *Echinococcus spp*., *Taenia spp*.), nematodes (ex. *Strongyloides spp*., *Toxocara*, *Trichinella*), and trematodes (ex. *Fasciola spp*., *Clonorchis)* as well as Pentastomida (ex. *Linguatula serrata*); Fungi, including microsporidia (ex. *Enterocytozooan bieneusi*, *Encephalitozoon cuniculi)*; and Protista, including protozoa (ex. *Giardia spp*., *Cryptosporidium spp*.). Food products can be parasitically tainted on both their exterior, such as with unwashed produce, or their interior, as with the infected flesh of meat/fish or dairy products [8,10–12]. Drinking water and recreational water can also serve as exposure pathways for acquiring enteric parasites as can the unintentional consumption of infected soil or parasitic material from items or objects, including animal fur, feathers or skin [13–17].

Human contact with the environment and animals has consistently evolved throughout history leading to varied ZEP risks and disease patterns among different population groups [4, 18]. Although early human civilizations lead transhumant lifestyles, this existence is much less common today as urban cities continue to expand, traditional migratory patterns are disrupted, environmental degradation changes the landscape, and governments incentivize more sedentary lifestyles [19]. However, several cultures continue to practice pastoralism as animal herders or nomads [19–22]. Nomadic and pastoral communities present unique challenges related to ZEPs due to their animal husbandry and contact, personal hygiene behaviors, diet and cooking methods, and water and sanitation utilization [20–22]. These families typically have close and frequent human-animal contact, lack improved water sources and sanitation infrastructure, and have hindered access to human health care facilities or veterinary care [21–24]. The purpose of this systematic review was to determine zoonotic enteric parasites and among nomadic and pastoralist people and examine the identified risk factors distinctive to this way of life. By gaining insight into the ZEPs of pastoralist communities, tailored One Health interventions can be developed to address the zoonotic enteric parasitic burden among these nomads, their animals, and their environment.

## Methods

**Fig 1:**
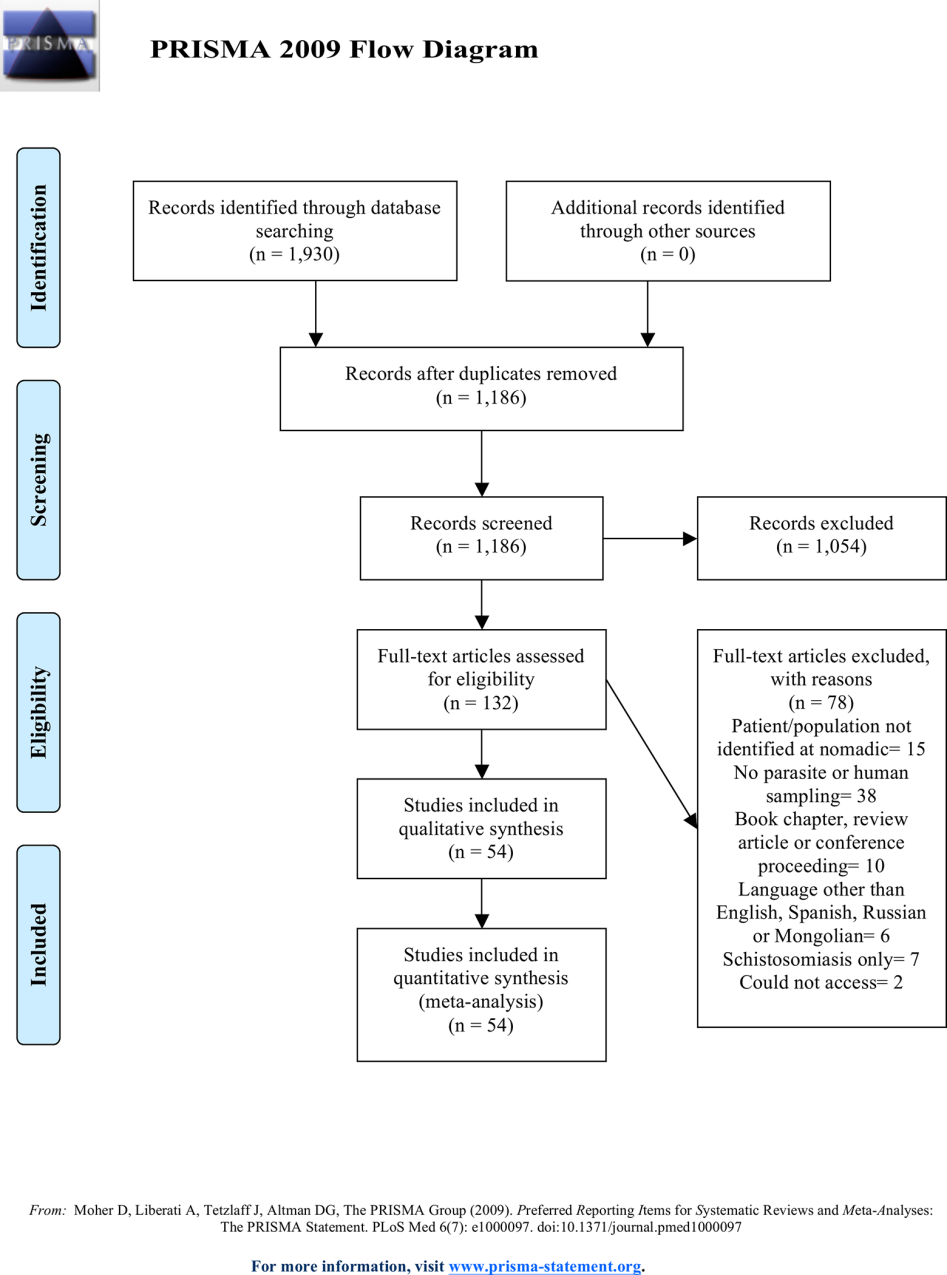
PRISMA flow diagram.

In performing this review we sought to follow the systematic review guidelines predefined by PRISMA [25]. In brief, a literature search **identified** possible articles for inclusion based on preset parameters and search terms. Next, the articles were **screened** for both duplicates and for topic. Then remaining articles were assessed for **eligibility** before **inclusion** in the final analysis. This process is illustrated through the PRISMA flow chart (Fig 1). Additional information can be found on the PRISMA checklist in the supplementary material (S1 Table).

### Criteria for inclusion

This review included journal articles with methods and results for the sampling of zoonotic enteric parasites among nomadic and pastoralist human populations. The list of zoonotic enteric pathogens used in this search was adapted from previous research and expanded by the authors (Table 1; S2 Table). Animal-only results were excluded as were studies with human sampling for non-enteric or non-zoonotic parasites and broad descriptions of the current health status of these groups. Conference proceedings, abstracts, book chapters, meeting notes, and editorial letters were also excluded. Journal articles were included for analysis if they were written in English, Spanish, Russian, or Mongolian due to the language abilities of the reviewers. The search was done for all published literature up until our final search date of November 29, 2016.

**Table 1:** Zoonotic enteric pathogens included in search by host and enteric risk factors for human transmission.

Type	Parasitic zoonosis	Synonyms and related terms	Pathogen name	Definitive host(s)	Intermediate host(s)	Enteric risk factors for human transmission
Cestode[26]	Alveolar echinococcosis	Alveolar hydatidosis	*Echinococcus multilocularis*	Foxes, Canids, and Cats	Small rodents	Ingestion of contaminated food, water, or soil
	Cystic echinococcosis	Hydatid disease	*Echinococcus granulosus*	Dogs; Other canids	Sheep, goats, swine, cattle, horses, and camels	
		Hydatidosis	Hydatid cyst			
	Cysticercosis	Neurocysticercosis	*Taenia solium*	Pigs	-	Ingestion of contaminated and undercooked pork
	Diphyllobothriosis	Diphyllobothriasis	*Diphyllobothrium*	Humans and other mammals	1) Copepods; 2) Minnows, larger fish	Ingestion of contaminated raw or undercooked seafood
		Bothriocephalosis	*Bothriocephalus*			
		Bothriocephaliasis	Broad tapeworm			
			Fish tapeworm			
	Sparganosis	Spirometrosis	*Spirometra*	Dogs and cats	1) Copepods; 2) Fish, reptiles, amphibians	Ingestion of contaminated water or amphibians/snakes
			*Sparganum*			
	Taeniosis	Taeniasis	*Taenia*	Humans	Cattle and pigs	Ingestion of contaminated and undercooked beef or pork
		Tapeworm				
Fungi[27]	Zoonotic microspora	Microsporidia	*Enterocytozooan bieneusi*	_	Pigs, macaques, horses, cats, rabbits, small rodents, reptiles, foxes, chickens, pigeons goats, cattle, donkeys, fish, and gorillas,	Likely ingestion of contaminated water or food
			*Encephalitozoon cuniculi*			
			*Encephalitozoon intestinalis*			
			*Encephalitozoon hellem*			
			`Pleistophora-like			
			organisms'			
Nematode[26,28–30]	Angiostrongylosis	Angiostrongyliasis	*Angiostrongylus cantonensis*	Rats	Snails; Slugs	Ingestion of contaminated and uncooked snails, slugs, shrimp, or crabs or unwashed produce contaminated from infected snails/slugs
	*Anisakidae* infections	–	*Anisakis*	Marine mammals	Crustaceans, fish, and squid	Ingestion of contaminated and undercooked marine fish
			*Pseudoterranova*			
	Capillariosis	Capillariasis	*Capillaria*	Fish-eating birds; Rodents	1) Freshwater fish; 2) Rodents, pigs, carnivores, and primates	Ingestion of contaminated and undercooked freshwater fish; ingestion of contaminated soil, water, or food
	Gnathostomosis	Gnathostomiasis	*Gnathostoma*	Pigs, cats, dogs, and other wildlife	Crustaceans, fish, frogs, snakes, and birds	Ingestion of contaminated water or undercooked fish/poultry
	Toxocarosis	Toxocariasis	*Toxocara*	Dogs	Small mammals	Ingestion of contaminated soil or small mammals
		Toxocariosis				
		Larva migrans				
	Trichinellosis	Trichinosis	*Trichinella*	Pigs and bears	Small rodents	Ingestion of contaminated and undercooked meat
	Zoonotic intestinal helminth infection	Helminthosis	Helminth	Humans, pigs, and primates; Cats and dogs; Birds, reptiles, amphibians, and other canids	Humans and pigs	Ingestion of contaminated soil or food
		Helminthiasis	*Ascaris*			
		Ascarosis	*Ancylostoma*			
		Ascariasis	Hookworm			
		Ancylostomosis	*Trichuris*			
		Ancylostomiasis	*Strongyloides*			
		Trichuriosis	*Alaria*			
		Trichuriasis	rat lungworm			
		Strongyloidosis	*Echinostoma spp*.			
		Strongyloidiasis	*Lagochilascaris minor*			
Protozoa[26, 31–35]	Toxoplasmosis	TORCH	*Toxoplasma*	Cats and other felidae	Birds and rodents; Livestock and wildlife	Ingestion of contaminated soil, water or food; ingestion of contaminated and undercooked meat
	Zoonotic intestinal protozoal infection	Protozoosis	Protozoa	Humans, primates, livestock, cats, dogs, wild mammals, birds, rodent, horses, reptiles, and amphibians	Cattle and pigs	Ingestion of contaminated food or water or undercooked meat
		Protozoasis	*Giardia*			
		*Giardiosis*	*Cryptosporidium*			
		*Giardiasis*.	*Blastocystis*			
		Cryptosporidiosis	*Sarcocystis*			
		Blastocystosis	*Cyclospora cayetanensis*			
		Sarcocystosis	*Entamoeba histolytica*			
		Cyclosporiasis	*Balantidium coli*			
		Cyclospora				
		Amoebiasis				
		Amoebic dysentery				
		Entamoeba				
		Balantidosis				
	Zoonotic trypanosomosis	Trypanosomiasis	*Trypanosoma cruzi*	Humans and other mammals	Triatomine bug	Ingestion of contaminated fruit juices or contaminated food by infected insects
		Chagas				
Trematode[36–37]	Foodborne trematodosis	Trematodiasis Fasciolosis	Fluke Trematode	Cats, dogs, foxes, pigs, and other ruminants	1) Snail; 2) Fish, mollusks, crustaceans, amphibians, and insects	Ingestion of contaminated and undercooked freshwater fish, crustaceans, aquatic plants, or tadpoles or snails or ingestion of contaminated water
		Fascioliosis	*Fasciola spp*.			
		Fasciolasis	*Fasciolopsis*			
		Fascioliasis	*Opisthorchis*			
		Distomatosis	*Clonorchis*			
		Fasciolopsosis	*Paragonimus*			
		Fasciolopsiosis	Minute intestinal fluke			
		Opisthorchosis	*Haplorchis pumilio*			
		Opisthorchiasis	*Metagonimus yokogawai*			
		Clonorchiosis	*Heterophyes spp*.			
		Clonorchiasis				
		Paragonimosis				
		Paragonimiasis				
		Metagonimus				
		Heterophyiasis				
		Heterophyiasis				
Tongue Worm[38]	Zoonotic pentastomes	Pentastomiasis	*Armillifer armillatus*	Snakes and reptiles	Dogs, foxes, wolves, and rodents	Ingestion of contaminated and undercooked snake meat or ingestion of contaminated food/water
		Linguatulosis	*Armillifer moniliformis*			
			*Linguatula serrata*			

Note: Pathogen list adapted from previous research [10–11, 20, 38–40]

### Search strategy for study identification

This search was conducted through the online databases PubMed, Web of Science (Core Collection, Zoological Record, Cabi, and Biosis), and twelve databases within Proquest (Agricultural Science Collection including Agricola, ProQuest Aquatic Science Collection‎, ProQuest Biological Science Collection‎, ProQuest Earth Science Collection‎, ProQuest Environmental Science Collection‎, COS Conference Papers Index‎, Health & Safety Science Abstracts‎, MEDLINE‎, and TOXLINE‎). Search strings were developed to search the title and abstract of publication for each zoonotic enteric parasite using the parasite name, known synonyms, and the name of any causative species. These pathogen strings were combined with key words for nomadic populations using Boolean Operators and wildcard symbols (*) such as:

1. “Alveolar echinococcosis”[tiab] OR “Alveolar hydatidosis”[tiab] OR “Echinococcus multilocularis”[tiab])
  AND
2. (nomad*[tiab] OR nomadic[tiab] OR pastoralis*[tiab] OR herder*[tiab] OR “semi-nomadic”[tiab] OR pastoral[tiab] OR nomadism[tiab] OR transhumance[tiab] OR transhumant[tiab] OR agropastoralist*[tiab] OR “agro-pastoralist”[tiab] OR “agro-pastoralists”[tiab])

The zoonotic enteric parasite search strings were then combined using OR to search for all of the key parasites at once AND pastoralist populations as references in either the title or abstract of the paper. A complete list of search terms and keywords and the search strings used for each database is listed in S2 and S3 Tables of the Supporting Information.

### Data screening

The primary author read through the titles and abstracts of the full list of retrieved articles and kept those that either a) demonstrated zoonotic enteric parasites in nomadic human populations; or b) the purpose and results of the article could not be determined based on title or abstract alone. When the adequacy of an article could not be determined by the abstract alone, full text versions were obtained. Complete articles were read by three reviewers and included in the final analysis based on the initial criteria and a majority decision. At this time, studies that involved *Schistosoma spp*. alone were discarded as the reviewers determined that it was not a true zoonotic enteric parasite based on transmission methods. Although included in systematic reviews of zoonotic enteric parasites by previous authors, further investigation into the transmission of *Schistosoma spp*. showed that the parasite must *penetrate* the skin and therefore enteric exposure by itself is not sufficient for infection [41].

## Results

Based on the initial search, 1,930 articles were selected across the multiple databases (Fig 1). Of these, 744 were duplicates and removed. From the remaining 1,186 articles, only 132 met the criteria for full-text consideration based on title and abstract or the content of the article could not be ascertained without a review. Articles were then excluded based on language other than English, Spanish, Russian or Mongolian, the paper was solely on *Schistosomiasis*, the full text could not be accessed, the material was not a journal article (ex. conference proceeding or book chapter), the study did not involve parasite or human sampling, or the study population was not identified as nomadic/pastoralist at the time of the study.

**Fig 2:**
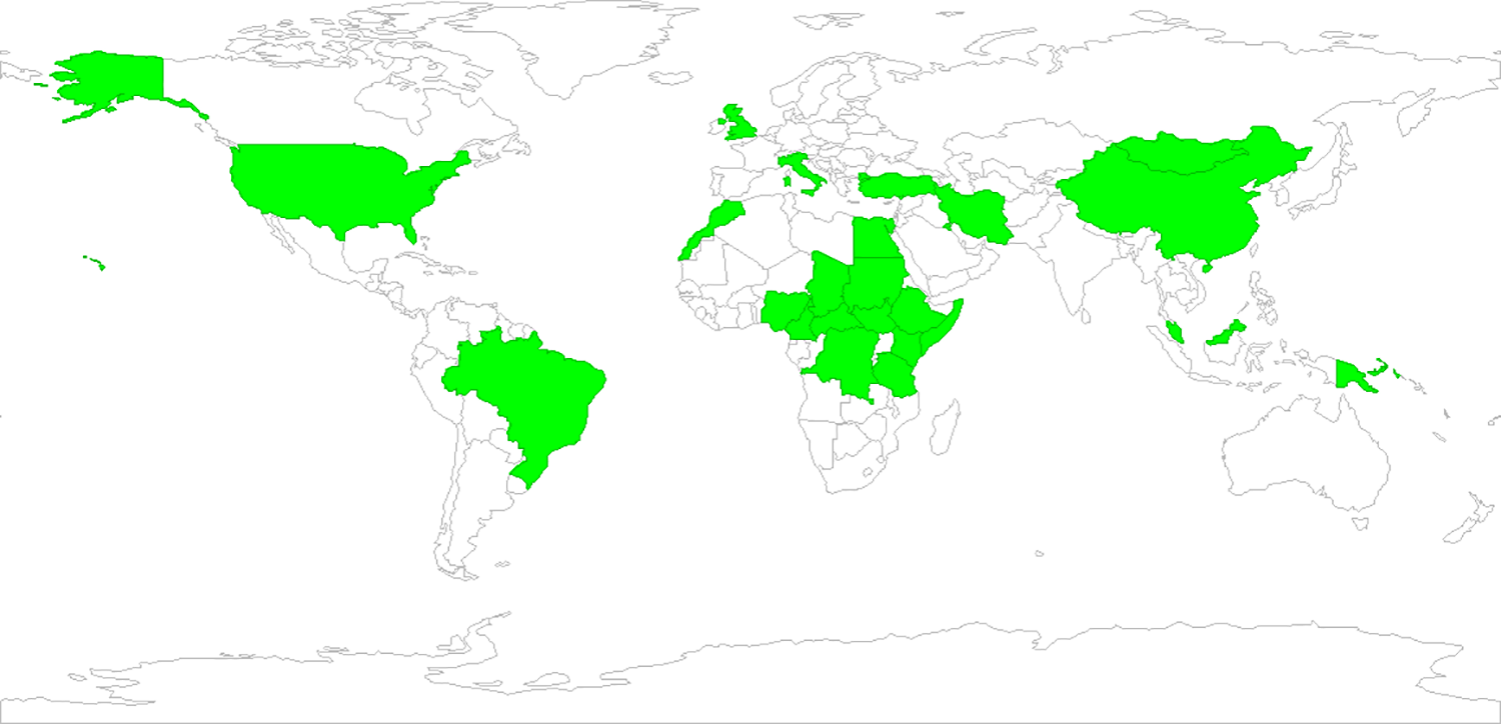
Included study sites by country using GeoDa software, version 1.10.0.8.

The range for publication dates spanned from 1956 through 2016 with research conducted as early as 1946 and as late as 2016. Research on zoonotic enteric parasites was performed on either humans alone or humans and domestic animals. Specimens collected included blood/serum, urine, stool, radiograph (x-ray) and ultrasound images, and patient medical records. Research was carried out in 24 countries among nomads, pastoralists, herders, and traveling people across a wide range of ZEPs (Fig 2).

### Zoonotic enteric parasites included in review

The included articles for this review found cestodes, nematodes, trematodes, and protozoa among many groups of nomadic and semi-nomadic people stretching across all continents except for Antartica (Table 2). In addition to the pathogens of the initial search, the enteric parasites of *Hymenolepsis* spp., *Trichomonas instestinalis* (*Pentatrichomonas hominis*), *Dirocoeliasis*, *Trichostongylus*, *Dientamoeba fragilis*, and *Dirofilaria immitis* were found in the selected studies and have been shown to be zoonotic [42–46]. Almost half of all of the selected citations studied *Echinococcosis spp*. (n = 26). Many of the studies also included some sort of testing for livestock and domestic animals, most often household dogs. Methods for ZEP detection varied across egg counts, microscopy and floatation/sedimentation techniques, antibody and titer testing, hospital record review, sonography and radiology results, skin snips and tests, and PCR analysis. ZEPs were found in nomadic, herding or pastoralist household and community members, students, military and agricultural workers, immigrants, settled inhabitants, hunters and fishermen, patients and staff from hospitals and orphanages, slaughterhouse personnel and travelling people. ZEPs were discovered in women and men and spanned all ages with prevalence rates between the groups dependent upon the pathogen and relevant exposure risks.

**Table 2:** Characteristics of studies included in systematic review.

Population	Study Country	Zoonotic Enteric Parasite(s)	Risk Factors	Year of Research	Citation
Semi-nomadic people	Turkey	*Entamoeba histolytica*	1. Livestock Contact	1954	Wells (1956)
		*Giardia spp*.			
		*Ancylostoma duodenale*			
		*Ascaris lumbricoides*			
		*Trichuris trichiura*			
		*Taenia spp*.			
Turkana and Massai pastoralists	Kenya	*Echinococcus granulosus*	1. Dog Contact/Feeding Dogs Offal	1952–1955	Wray (1958)
Bedouin nomads and immigrants	Kuwait	*Echinococcus granulosus*	1. WASH	1956–1960	Aly el Gazzar & McCreapie (1962)
			2. Dog Contact/Feeding Dogs Offal		
			3. Livestock Contact		
			4. Food Handling		
Bedouins nomads	Egypt	*Ascaris lumbricoides*	-	1962	Van Peenen & Reid (1963)
		*Ancylostoma duodenale*			
		*Entamoeba histolytica*,			
		*Trichuris trichiura*			
Agricultural workers and nomadic herders	Somalia	*Echinococcus granulosus*	1. Dog Contact/Feeding Dogs Offal	1968*	Kagan & Cahill (1968)
		*Dirofilaria immitis*	2. Livestock Contact		
		*Entamoeba histolytica*	3. Butchering/Slaughtering		
		*Toxoplasma gondii*			
Nomadic and settled Hadza people	Tanzania	*Toxoplasma gondii*	1. WASH	1966–1967	Bennet et al. (1970)
		*Entamoeba histolytica*	2. Dog Contact/Feeding Dogs Offal		
		*Trichinella*	3. Wildlife Contact		
		*Dientamoeba fragilis*	4. Butchering/Slaughtering		
		*Giardia spp*.	5. Consumption of Raw/Undercooked Meat		
		*Trichuris trichiura*	6. Food Handling		
		*Ascaris spp*.	7. Housing Structure		
		*Fasciola spp*.			
		*Taenia spp*.			
Nomadic Babinga, Bayaka and Badjelli people	Central African Republic, Cameroon and Ethiopia	*Strongyloides spp*.	-	1968–1969	Pampiglione & Ricciardi (1971)
Immigrants from Zabol	Iran	*Ascaris spp*.	1. Age (Children under 14)	1973*	Ghadirian & Missaghian (1973)
		*Trichuris spp*.			
		*Trichostrongylus spp*.			
		*Hymenolepsis nana*			
		Hookworm spp.			
Nomadic and settled Mongolian herders	Mongolia	*Echinococcus multilocularis*	1. Dog Contact/Feeding Dogs Offal	1969	Jezek et al. (1973)
			2. Livestock Contact		
			3. Age (Adults)		
Nomadic and settled people	Iran	*Trichostrongylus spp*.	1. Livestock Contact	1974*	Ghadirian, Arfaa, & Sadighian (1974)
			2. Housing Structure		
Nomadic Bakhtiari people	Iran	*Ascaris spp*.	1. Livestock Contact	1973	Ghadirian, Arfaa, & Arvanaghi (1974)
		*Trichostrongylus spp*	2. Housing Structure		
		*Trichuris trichiura*,			
		*Hymenolepsis nana*			
		.*Taenia saginata*			
Nomadic Babinga people	Central African Republic	*Trichuris trichiura*	1. WASH	1968–1970	Pampiglione & Ricciardi (1974)
		Ancylostoma lumbricoides	2. Dog Contact/Feeding Dogs Offal		
		*Strongyloides spp*.	3. Wildlife Contact		
		*Entamoeba histolytica*	4. Consumption of Raw/Undercooked Meat		
		*Giardia spp*.			
		*Toxoplasma gondii*			
		*Toxocara spp*			
		*Trichomonas instestinalis*			
		*Dientamoeba fragilis*			
Nomadic Bakhtiari people	Iran	*Trichostrongylus spp*.	1. Livestock Contact	1967–1974	Ghadirian & Arfaa (1975)
			2. Food Handling		
			3. Housing Structure		
Hausa, Fulani, Gungawa, Kambari, Dukawa and Sarkawa people	Nigeria	Hookworm spp.	1. Livestock Contact	1970	Oomen (1975)
		*Entamoeba histolytica*	2. Recreational/Environmental Water Contact		
Bambuti people	Democratic Republic of the Congo	*Entamoeba histolytica*	1. WASH	1971–1972	Pampiglione et al (1979)
		*Giardia spp*.	2. Wildlife Contact		
		*Dientamoeba fragilis*	3. Butchering/Slaughtering		
		Hookworm spp.	4. Food Handling		
		*Trichuris trichiura*	5. Age (Children)		
		*Strongyloides spp*.	6. Housing Structure		
		*Ascaris lumbricoides*			
Nomadic and settled people	Sudan	*Taenia saginata*	1. WASH	1980*	Bella et al. (1980)
		*Hymenolepsis nana*	2. Recreational/Environmental Water Contact		
		*Ancylostoma duodenale*	3. Housing Structure		
		*Strongyloides stercoralis*			
Seminomadic pastoralists and settled people	Ethiopia	*Ascaris lumbricoides*	1. WASH	1981*	Kloos, Desole, & Lemma (1981)
		*Trichuris trichiura*	2. Livestock Contact		
		Hookworm spp.	3. Housing Structure		
		*Strongyloides spp*.			
		*Hymenolepsis spp*.			
		*Taenia spp*.			
		*Entamoeba histolytica*			
		*Giardi spp*.			
		*Fasciola spp*.			
		*Balantidium coli*			
Mormon herders	United States of America	*Echinococcus granulosus*	1. Dog Contact/Feeding Dogs Offal	1946–1980	Crellin et al. (1982)
			2. Livestock Contact		
Nomadic and semi-nomadic people	Somalia	*Ascaris lumbricoides*	1. WASH	1987*	Ilardi et al. (1987)
		*Ancylostoma duodenale*	2. Livestock Contact		
		*Trichuris trichiura*	3. Consumption of Raw/Unprocessed Milk		
		*Giardia spp*.	4. Recreational/Environmental Water Contact		
		*Toxoplasma gondii*			
Travelling people	Scotland	*Toxoplasma gondii*	1. WASH	1987*	Jackson, Hutchison, & Siim (1987)
			2. Consumption of Raw/Undercooked Meat		
			3. Food Handling		
Turkana people	Kenya	*Echinococcus granulosus*	1. Sex (Women)	1985	MacPherson et al. (1987)
Nomadic people	Papua New Guinea	*Strongyloides spp*.	1. Livestock Contact	1983–1985	Barnish & Ashford (1989)
			2. Age (children)		
Purko people	Tanzania	*Echinococcus granulosus*	1. Dog Contact/Feeding Dogs Offal	1985	Macpherson et al (1989)a
			2. Livestock Contact		
			3. Butchering/Slaughtering		
			4. Consumption of Raw/Unprocessed Milk		
			5. Consumption of Raw/Undercooked Meat		
			6. Sex/Gender (Women)		
Turkana, Nyangatom, Boran and Maasai people	Kenya, Sudan, Ethiopia and Tanzania	*Echinococcus granulosus*	1. WASH	1985–1987	Macpherson et al. (1989)b
			2. Dog Contact/Feeding Dogs Offal		
			3. Livestock Contact		
			4. Butchering/Slaughtering		
			5. Consumption of Raw/Unprocessed Milk		
			6. Consumption of Raw/Undercooked Meat		
			7. Sex/Gender (Women)		
Nomadic shepherds	Iran	*Cryptosporidium spp*.	1. Livestock Contact	1990	Nouri & Karami (1991)
Pastoral and settled herders	China	*Echinococcus granulosus*	1. Livestock Contact	1993*	Chai (1993)
		*Taenia saginata*			
Tukano and Maku people	Brazil	Hookworm spp.	1. Dog Contact/Feeding Dogs Offal	1978	Chernela & Thatcher (1993)
		*Trichuris trichiura*	2. Wildlife Contact		
		*Ascaris lumbricoides*			
		*Entamoeba histolytica*			
		*Giardia spp*.			
		*Balantidium coli*			
		*Strongyloides stercoralis*			
Hamar pastoralists	Ethiopia	*Echinococcus granulosus*	1. Livestock Contact	1989	Klungsoyr, Courtright, & Hendrikson (1993)
			2. Wildlife Contact		
			3. Consumption of Raw/Unprocessed Milk		
			4. Age (Adults) & Sex/Gender (Women)		
Turkana and Massai pastoralists	Kenya	*Entamoeba histolytica*	1. WASH	1991	Harragin (1994)
		*Echinococcus spp*.	2. Dog Contact/Feeding Dogs Offal		
			3. Livestock Contact		
			4. Age (Children) & Sex/Gender (Women)		
Turkana nomads	Kenya	*Toxocara spp*.	1. WASH	1995*	Kenny et al. (1995)
			2. Dog Contact/Feeding Dogs Offal		
			3. Recreational/Environmental Water Contact		
			4. Housing Structure		
Nomadic and settled herders	Mongolia	*Echinococcus granulosus*	1. Dog Contact/Feeding Dogs Offal	1997*	Watson-Jones et al (1997)
			2. Livestock Contact		
			3. Butchering/Slaughtering		
Semi-nomadic Tibetan people	China	*Echinococcus multilocularis*	1. Dog Contact/Feeding Dogs Offal	1956–1997	Zhou et al. (2000)
			2. Wildlife Contact		
			3. Sex/Gender (Women)		
Semi-nomadic people	China	*Echinococcus granulosus*	1. Dog Contact/Feeding Dogs Offal	1990–1999	Wang et al. (2001)
			2. Livestock Contact		
			3. Butchering/Slaughtering		
			4. Age (Adults)		
Semi-nomadic people	Malaysia	*Trichuris trichiura*	1. Age (Children) & Sex/Gender (Women)	2002*	Sagin et al. (2002)
		*Ascaris lumbricoides*			
		*Giardia spp*.			
		*Hymenolepsis nana*			
Nomadic shepherds and butchers	Egypt	*Dicrocoelium spp*.	1. WASH	2003*	Haridy et al. (2003)
			2. Livestock Contact		
			3. Butchering/Slaughtering		
			4. Consumption of Raw/Undercooked Meat		
			5. Food Handling		
Semi-nomadic Tibetan people	China	*Echinococcus spp*.	1. WASH	1997–1998	Schantz et al. (2003)
			2. Dog Contact/Feeding Dogs Offal		
			3. Livestock Contact		
			4. Age (Adults) & Sex/Gender (Women)		
Fulani people	Nigeria	*Ascaris lumbricoides*	1. WASH	2003–2004	Anosike et al. (2004)
		Hookworm spp.	2. Livestock Contact		
		*Strongyloides stercoralis*	3. Recreational/Environmental Water Contact		
		*Trichuris trichiura*	4. Housing Structure		
		*Entamoeba histolytica*			
Berber people	Morocco	*Echinococcus granulosus*	1. Dog Contact/Feeding Dogs Offal	2000–2001	Macpherson et al. (2004)
			2. Livestcok Contact		
			3. Butchering/Slaughtering		
			4. Sex/Gender (Women)		
Semi-nomadic Tibetan people	China	*Echinococcus multilocularis*	1. Housing Structure	2001–2002	Wang et al. (2004)
Semi-nomadic Tibetan people	China	*Echinococcus spp*.	1. WASH	2000–2001	Li et al. (2005)
			2. Dog Contact/Feeding Dogs Offal		
			3. Livestock Contact		
			4. Wildlife Contact		
			5. Food Handling		
			6. Age (Adults) & Sex/Gender (Women)		
Maasai people	Tanzania	*Ancylostoma duodenale*	1. WASH	2005*	Nyaruhucha, Mamiro, & Kerengi (2005)
		*Ascaris lumbricoides*	2. Livestock Contact		
		*Trichuris trichiura*			
Camel herders	Sudan	*Toxoplasma gondii*.	1. Livestock Contact	2007*	Khalil et al. (2007)
			2. Consumption of Raw/Unprocessed Milk		
			3. Consumption of Raw/Undercooked Meat		
Nomadic families	Iran	*Echinococcus granulosus*	1. Dog Contact/Feeding Dogs Offal	2001–2003	Rafiei et al. (2007)
			2. Livestock Contact		
			3. Butchering/Slaughtering		
Semi-pastoralist Kara and Kwego people	Ethiopia	*Entamoeba histolytica*	1. WASH	2006	Teklehaymanot (2009)
		*Giardia spp*.	2. Livestock Contact		
		*Ascaris lumbricoides*			
		*Trichuris trichiura*			
		Hookworm spp.			
		*Strongyloides stercoralis*			
Pastoralists	Italy	*Echinococcus granulosus*	1. Livestock Contact	2001–2005	Conchedda et al. (2010)
			2. Age (adults) & Sex/Gender (Men)		
Fulani people	Nigeria	*Ascaris lumbricoides*	1. WASH	2009	Jombo et al. (2010)
		Hookworm spp.	2. Sex (Males)		
		*Strongyloides stercoralis*	3. Housing Structure		
		*Trichuris trichiura*			
		*Entamoeba histolytica*			
Semi-nomadic Tibetan people	China	*Echinococcus spp*.	1. WASH	2001–2008	Li et al. (2010)
			2. Dog Contact/Feeding Dogs Offal		
			3. Livestock Contact		
			4. Wildlife Contact		
			5. Age (Adults) & Sex/Gender (Women)		
Mongolian herders	China	*Echinococcus granulosus*	1. Dog Contact/Feeding Dogs Offal	1995–1996	WenBin et al. (2011).
			2. Livestock Contact		
			3. Butchering/Slaughtering		
Pastoralist Foulbe and Arabic and settled people	Chad	*Ascaris lumbricoides*	1. WASH	2008	Bechir et al. (2012)
		*Entamoeba histolytica*	2. Livestock Contact		
		Hookworm spp.	3. Consumption of Raw/Undercooked Milk		
		*Taenia saginata*	4. Consumption of Raw/Undercooked Meat		
		*Hymenolepsis nana*	5. Age (children) & Sex/Gender (Women)		
		*Giardia spp*.			
		*Trichomonas instestinalis*			
Semi-nomadic Tibetan people	China	*Echinococcus spp*.	1. WASH	2007; 2009	Giordani et al. (2012)
		*Ascaris spp*.	2. Dog Contact/Feeding Dogs Offal		
			3. Livestock Contact		
			4. Butchering/Slaughtering		
			5. Consumption of Raw/Unprocessed Milk		
			6. Consumption of Raw/Undercooked Meat		
			7. Recreational/Environmental Water Contact		
Turkana nomads	Kenya	*Echinococcus granulosus*	1. Sex/Gender (Women)	2013*	Mutwiri et al. (2013)
Mundari pastoralists	South Sudan	*Echinococcus granulosus*	1. Dog Contact/Feeding Dogs Offal	2013*	Stewart et al. (2013)
			2. Livestock Contact		
			3. Butchering/Slaughtering		
			4. Sex/Gender (Women)		
Nomadic and settled people	Egypt	*Cryptosporidium spp*.	1. Dog Contact/Feeding Dogs Offal	2013	Awadallah & Salem (2015)
		*Ascaris lumbricoides*	2. Livestock Contact		
		*Heterophyes spp*.	3. Food Handling		
		*Ancylostoma spp*.			
		*Paragonimus spp*.			
		*Hymenolepis nana*			
		*Toxocara spp*.			
Behbahan nomads	Iran	*Echinococcus granulosus*	1. WASH	2015–2016	Kasaei, Tavalla, & Etebar (2016)
			2. Dog Contact/Feeding Dogs Offal		
			3. Livestock Contact		
			4. Food Handling		

*Study date not listed in methods; WASH = water, sanitation, and hygiene

### Identified risk factors for nomadic/pastoralist populations

Several risk factors were found in the participating nomadic or pastoralist communities across the different studies (Table 2). These exposure hazards can be grouped by animal contact, food preparation and diet, and household characteristics. For example, animal contact among nomadic and pastoralist communities with ZEP ranged from close physical contact and shared housing to simply allowing nearby wildlife to interact with domestic animals [47–51]. Dog contact and/or ownership was a primary risk factor across multiple ZEP pathogens and the risk for infection and zoonotic disease transmission increased when dogs were fed the raw offal or viscera of slaughtered livestock or fish [52–57]. However, contact with livestock on the whole was also associated with ZEP infection among the study participants [58–60]. Several groups also have significant contact with wildlife either from their location near forested areas or from hunting bush meat, rodents, birds, or through fishing and seafood harvesting [61–65].

ZEP risk factors were presented in the results of the citations that were the result of food acquisition, preparation, and consumption trends. For instance, home butchering and slaughtering of livestock and/or wild game was associated with ZEP prevalence among some nomadic groups [66–70]. Additionally, not washing or cleaning food properly prior to cooking was identified as a risk factor in several studies [71–73]. Dietary trends and practices associated with the consumption of raw or unprocessed/undercooked milk products and/or meat left several pastoralist communities at risk for procuring ZEPs [74–77].

Finally, some ZEP risk factors recognized by the collection of research articles centered on the roles or responsibilities of household members from nomadic families and housing characteristics [78–85]. The household’s access to adequate water, sanitation, and hygiene behaviors (WASH) influenced ZEP infection [86–89]. Aside for drinking water sources, contact with environmental water sources and even housing construction were also associated with ZEP transmission [72,90–94]. Cultural, ethnic, religious, and geographical differences between the nomadic populations presented in this review offer even greater variance of threats for infection with a zoonotic enteric parasite [53,95–99].

## Discussion

While some zoonoses exposure risks are associated with rural living or animal husbandry in general, the close association and proximity between nomadic people and domestic animals introduces a unique human-animal interface that may present even greater One Health challenges for ZEP prevention. There are an estimated 180 million pastoralists across the world and the competition for resources, particularly water, is leading to increased and intensified exchanges between people, domestic animals, and wildlife in nomadic areas [100]. These interactions escalate the exposure risks for zoonotic and reverse zoonotic disease among each group.

When examining the category of animal contact as a risk factor for ZEP transmission among nomadic pastoralist populations, dogs were present or owned by the majority of the participants studied across the included articles and served as guards for livestock, as hunting assistance, and as companions [22]. Several zoonotic enteric parasites can be transmitted to humans from dogs, cats, and other pets/companion animals [101]. In this review, many of the study authors pointed out that interactions with dogs, in particular, are a high risk for ZEP transmission among nomadic and pastoralist communities largely due to the practice of throwing viscera and offal from slaughtered animals to the dogs to eat [51,54–57,59,61,62,64–66,68–71,73,89,91,98–99,102]. For example, this behavior is estimated to increase the exposure risk for acquiring *Echinococcosus spp*. by almost five times as compared to people who do not feed offal to dogs [103]. Additional ZEPs such as *Toxoplasma spp*.and *Toxocara spp*., can be transmitted to dogs or cats through the ingestion of infected meat or viscera which can then expose humans due to their close association with humans [104].

Cohabitation with dogs and other livestock in homes, huts, or tents was common in several participating study households [48–49,52,56,67,73]. In one instance, researchers found that almost all of the participating pastoralists reported sharing familial cooking pots with dogs while in other nomadic societies of the studies presented, researchers noted that dogs were used to clean up the waste and vomit of children [47,50,52,70]. This demonstrates an intimacy shared between dogs and nomadic and pastoralist communities but also illustrates the threat of ZEP exposure between humans and animals.

Aside from dogs, nomads and pastoralists have significant animal contact through their work with livestock and interactions with wildlife. Herding animals across large ranges and handling animals for food products means close contact with livestock. Many nomadic and pastoralist communities utilize every part of the animal. Pastoral households often dry animal manure to use for heating and cooking and may use animal hair or hides for clothing or tents [49,65,67,79]. Because of their mobility, dead members of the community are usually not buried but instead fed to local carnivores [21,47]. Wildlife share the same space as the pastoral communities in many regions and due to their bounty and diversity, ZEPs are provided multiples opportunities for intermediate and definitive host species for which to proliferate [21]. Some nomadic communities also hunt wildlife leading to more exposure threats for ZEP transmission to humans [47,51,61,62,65,67,81,64–65].

Food preparation and diet creates multiple opportunities for ZEP exposure, particularly among nomadic communities. [4,8,18,21]. As a primary source of nutrition through meat, milk and even blood products, animals serve as a lifeline to the dietary needs of many pastoralist societies [21–22]. However, the consumption of raw or undercooked meat and organs or unprocessed milk and blood was noted as potential vehicles for ZEP transmission among nomadic groups from the included studies of this review [50,52–53,61–62,65,74–77,84,86,92]. Pastoralists and nomads who also eat raw or undercooked snails, fish, reptiles, or amphibians or those who consume insects such as ants either intentionally or unintentionally are at risk for infection with multiple ZEPS as well [63–64,67,72–73].

Aside from eating or drinking contaminated food items, preparation methods prior to consumption can also expose nomadic and pastoralist households to ZEPs. Home slaughter of livestock, wildlife, small rodents, fish, birds, reptiles, and amphibians have the potential to introduce zoonotic parasites from the infected exterior and interiors of the animals through accidental ingestion or inhalation during the butchering process [50–53,55,57,61,65–70,91,99,102]. But it isn’t just flesh or animal products that put humans at risk for ZEP transmission. Unwashed vegetables and fruits were also noted as an exposure threat for participating nomadic communities across the included studies [67,71–73,79,86,89].

The defined roles and responsibilities of household members, residential infrastructure, and water, sanitation, and hygiene within pastoralist communities can also introduce ZEP threats. Although all members of pastoral families have chores and tasks related to their communal well being, some jobs appear heavily along gender lines. For example, hunting, herding livestock to water and seasonal pastoral lands, and slaughter tend to be male-dominated [24]. These activities take men away from the home and into the larger environment, where ZEPs in environmental water sources and wildlife may dominate. In contrast, women are in charge of most household work such as raising and rearing children, caring for the sick and old, collecting firewood or preparing animal dung, retrieving water, milking animals, preserving and preparing food, weaving items and clothing, and providing education to the children [24]. Nomadic women also care for and have more contact with dogs at the home, leading to higher rates of some ZEPs such as *Echinococcosus spp*. [21]. In the articles summarized by this review, males and females demonstrated differing levels of ZEP infection and demonstrated unique exposure risks associated with not only gender but also with age as children were more likely to engage in play with dogs or exhibit exploratory mouthing behaviors as toddlers [50,51–53,59,65,67,70,73,76,78, 82–85,87,91–92,94,99,102].

Water, sanitation, and hygiene (WASH) access and behaviors can greatly influence ZEP infections in nomads. A lack of proper hand washing behaviors, the failure to wash fruits and vegetables with clean water prior to eating, practicing open defecation near the camp/household, ritual or cultural use of animal products, and the recreational use of environmental water sources for drinking, bathing, laundry, watering animals, and fishing were noted as risk factors for zoonotic enteric parasite exposure among the included studies [47,54,61–62,65,67,69–75,79,84,86–92,94]. Housing type and structure may also play a part in the transmission of ZEPs to pastoral groups as animals and vectors can enter freely and exposure people, food, drinking water, and the home environment to parasites as highlighted in several studies [49,52,61,67,70,86,90,92–93].

Although this review examined risk factors related to ZEP infection among nomadic and pastoral populations by animal contact, food preparation and diet, and household characteristics, several areas of research were missing when attempting to describe ZEP exposure threats within transhumant societies. For example, specific cultural, ethnic or traditional customs and medicine can put certain nomadic groups at a higher risk for zoonotic parasite transmission than their sedentary neighbors or even nomadic counterparts from another region. These include ceremonial behaviors, dress, and foods, which are not highlighted by this study. Investigation into specific nomadic cultures should consider these additional risk factors and search literature and language explicit to the pastoralist group in question. Additionally, localized reports on ZEPs may have been left out of this review due to the parameters, terminology and databases used for the search.

Furthermore, any protective effects the nomadic way of life may provide against ZEP exposure are not considered. There are some studies that suggest a positive relationship between contact with livestock and the lower incidence of some ZEPs, such as with nomadic groups who consume a predominately milk diet exhibiting lower rates of *Entamoeba histolytica* infection or the fact that the pastoralist life of mobility means that the living space of the camps do not become overwhelmed with human and animal waste [21–22]. Further research into the relationship between nomadic societies and zoonotic enteric parasite should look at both risk factors and protective measures that are distinct to these communities and the cultural and ethnic identity of its inhabitants.

## Conclusion

Based on the acquired knowledge of this systematic review, the health of nomads and pastoralists is directly tied to the health of their livestock and surrounding environment. Future research on zoonotic enteric parasites or interventions to prevent their transmission to humans must be grounded in the One Health theory so that the multiple risk factors presented herein can be addressed. Nomadic and pastoral populations are a link to the past, present, and future of humans and the public health community should increase efforts to improve the health and well being of all global citizens. This will require tailored efforts to make animal contact safe for the pastoralists, decrease hazards related to food handling and preparation through access to WASH infrastructure and training, and addressing family dynamics which could be putting one group at a higher risk than another through education and awareness campaigns.

## Supporting information

S1 Table PRISMA checklist. (DOCX)

S2 Table Search terms by topic categories. (DOCX)

S3 Table Search strings per database and results from search of any time through November 29, 2016. (DOCX)
