# Supplementary material for: A systematic review of zoonotic enteric parasitic diseases among nomadic and pastoral people
Source: PLoS One. 2017 Nov 30;12(11):e0188809. doi: 10.1371/journal.pone.0188809 (PMC5708844; doi:10.1371/journal.pone.0188809)
Supplement: S2 Table — (DOCX) [file pone.0188809.s002.docx]

| S2 Table: Search terms by topic categories | | | |
| --- | --- | --- | --- |
| **Parasitic zoonosis** | **Synonyms and related terms** | **Pathogen name** | **Population** |
| Alveolar echinococcosis | Alveolar hydatidosis | *Echinococcus multilocularis* | nomad |
| Angiostrongylosis | Angiostrongyliasis | *Angiostrongylus* *cantonensis* | nomad |
| *Anisakidae* infections | – | *Anisakis*  *Pseudoterranova* | nomadic |
| Capillariosis | Capillariasis | *Capillaria* | pastoralist |
| Cystic echinococcosis | Hydatid disease  Hydatidosis | *Echinococcus granulosus*  Hydatid cyst | pastoralists |
| Cysticercosis | Neurocysticercosis | *Taenia solium* | herder |
| Diphyllobothriosis | Diphyllobothriasis  Bothriocephalosis  Bothriocephaliasis | *Diphyllobothrium*  *Bothriocephalus*  Broad tapeworm  Fish tapeworm | herders |
| Foodborne trematodosis | Trematodiasis  Fasciolosis  Fascioliosis  *Fasciola spp.*sis  Fascioliasis  Distomatosis  Fasciolopsosis  Fasciolopsiosis  Opisthorchosis  Opisthorchiasis  Clonorchiosis  Clonorchiasis  Paragonimosis  Paragonimiasis  Metagonimus  Heterophyiasis | Fluke  Trematode  *Fasciola spp.*  *Fasciolopsis*  *Opisthorchis*  *Clonorchis*  *Paragonimus*  Minute intestinal fluke  *Haplorchis pumilio*  *Metagonimus yokogawai*  *Heterophyes spp.* | pastoralism |
| Gnathostomosis | Gnathostomiasis | *Gnathostoma* | pastoral |
| Sparganosis | Spirometrosis | *Spirometra*  *Sparganum* | nomadism |
| Taeniosis | Taeniasis  Tapeworm | *Taenia* | semi-nomadic |
| Toxocarosis | Toxocariasis  Toxocariosis  Larva migrans | *Toxocara* | transhumance |
| Toxoplasmosis | TORCH | *Toxoplasma* | transhumant |
| Trichinellosis | Trichinosis | *Trichinella* | agropastoralist |
| Zoonotic intestinal protozoal infection | Protozoosis  Protozoasis  Giardiosis  *Giardia spp.*sis  Cryptosporidiosis  Blastocystosis  Sarcocystosis  Cyclosporiasis  Cyclospora  Amoebiasis  Amoebic dysentery  Entamoeba  Balantidosis | Protozoa  *Giardia spp.*  *Cryptosporidium*  *Blastocystis*  *Sarcocystis*  *Cyclospora cayetanensis*  *Entamoeba histolytica*  *Balantidium coli* | agro-pastoralist |
| Zoonotic schistosomosis | Schistosomiasis  Bilharziosis  Snail fever  Swimmers' itch | *Schistosoma*  *Bilharzia* |  |
| Zoonotic trypanosomosis | Trypanosomiasis  Chagas | *Trypanosoma cruzi* |  |
| Zoonotic intestinal helminth infection | Helminthosis  Helminthiasis  Ascarosis  Ascariasis  Ancylostomosis  Ancylostomiasis  Trichuriosis  Trichuriasis  Strongyloidosis  Strongyloidiasis | Helminth  *Ascaris*  *Ancylostoma*  Hookworm  *Trichuris*  *Strongyloides*  *Alaria*  rat lungworm  *Echinostoma spp.*  *Lagochilascaris minor* |  |
| Zoonotic microspora | Microsporidia | *Enterocytozooan bieneusi*  *Encephalitozoon cuniculi*  *Encephalitozoon intestinalis*  *Encephalitozoon hellem*  `Pleistophora-like  organisms' |  |
| Zoonotic pentasomes | Pentastomiasis  Linguatulosis | *Armillifer armillatus*  *Armillifer moniliformis*  *Linguatula serrata* |  |
| Zoonotic enteric pathogen |  |  |  |
| Zoonotic enteric parasite |  |  |  |
| Zoonotic parasite |  |  |  |
| animal-to-human enteric |  |  |  |
| foodborne parasite |  |  |  |
| waterborne parasite |  |  |  |
| enteric parasite |  |  |  |
| parasitism |  |  |  |

Note. Table adapted from 1) Devleesschauwer B, Ale A, Torgerson P, Praet N, de Noordhout CM, Pandey BD, Pun SB, Lake R, Vercruysse J, Joshi DD, Havelaar AH. The burden of parasitic zoonoses in Nepal: a systematic review. PLoS neglected tropical diseases. 2014 Jan 2;8(1):e2634; 2) Dorny P, Praet N, Deckers N, Gabriel S. Emerging food-borne parasites. Veterinary parasitology. 2009 Aug 7;163(3):196-206; 3) Slifko TR, Smith HV, Rose JB. Emerging parasite zoonoses associated with water and food. International journal for parasitology. 2000 Nov 30;30(12):1379-93; 4) Blench RM. Pastoralism in the new millennium, animal health and production series no. 150. FAO, Rome, Italy. 2001:1-06; and 5) Torgerson PR, Macpherson CN. The socioeconomic burden of parasitic zoonoses: global trends. Veterinary parasitology. 2011 Nov 24;182(1):79-95.
