# Supplementary material for: A systematic review of zoonotic enteric parasitic diseases among nomadic and pastoral people
Source: PLoS One. 2017 Nov 30;12(11):e0188809. doi: 10.1371/journal.pone.0188809 (PMC5708844; doi:10.1371/journal.pone.0188809)
Supplement: S3 Table — (DOCX) [file pone.0188809.s003.docx]

| S3 Table: Search strings per database and results from search of any time through November 29, 2016 | | |
| --- | --- | --- |
| **Database** | **Search String** | **Article Results** |
| Pubmed | ("Zoonotic enteric pathogen"[tiab] OR "Zoonotic enteric pathogens"[tiab] OR "Zoonotic enteric parasite"[tiab] OR "Zoonotic enteric parasites"[tiab] OR "Zoonotic parasite"[tiab] OR "Zoonotic parasites"[tiab] OR "Animal-to-Human parasite"[tiab] OR "Animal-to-Human parasites"[tiab] OR "Foodborne parasite"[tiab] OR "Foodborne parasites"[tiab] OR "Waterborne parasite"[tiab] OR "Waterborne parasites"[tiab] OR "Water-related parasite"[tiab] OR "Water-related parasites"[tiab] OR "Enteric parasite"[tiab] OR "Enteric parasites"[tiab] OR parasitism[tiab] OR "Zoonotic intestinal helminth infection"[tiab] OR "Zoonotic intestinal helminth infections"[tiab] OR Helminthosis[tiab] OR Helminthiasis[tiab] OR Ascarosis[tiab] OR Ascariasis[tiab] OR Ancylostomosis[tiab] OR Ancylostomiasis[tiab] OR Trichuriosis[tiab] OR Trichuriasis[tiab] OR Strongyloidosis[tiab] OR Strongyloidiasis[tiab] OR Helminth*[tiab] OR Ascaris[tiab] OR Ancylostoma[tiab] OR Hookworm*[tiab] OR Trichuris[tiab] OR Strongyloides[tiab] OR Alaria[tiab] OR "rat lungworm"[tiab] OR "rat lungworms"[tiab] OR "Echinostoma"[tiab] OR "Lagochilascaris minor"[tiab] OR "Zoonotic trypanosomosis"[tiab] OR Trypanosomiasis[tiab] OR Chagas[tiab] OR "Trypanosoma cruzi"[tiab] OR "Zoonotic schistosomosis"[tiab] OR Schistosomiasis[tiab] OR Bilharziosis[tiab] OR "Snail fever"[tiab] OR "Swimmer itch"[tiab] OR "Swimmers itch"[tiab] OR Schistosoma[tiab] OR Bilharzia[tiab] OR "Zoonotic intestinal protozoal infection"[tiab] OR "Zoonotic intestinal protozoal infections"[tiab] OR Protozoosis[tiab] OR Protozoasis[tiab] OR Giardiosis[tiab] OR Giardiasis[tiab] OR Cryptosporidiosis[tiab] OR Blastocystosis[tiab] OR Sarcocystosis[tiab] OR Cyclosporiasis[tiab] OR Cyclospora[tiab] OR Amoebiasis[tiab] OR "Amoebic dysentery"[tiab] OR Entamoeba[tiab] OR Balantidosis[tiab] OR Protozoa[tiab] OR Giardia[tiab] OR Cryptosporidium[tiab] OR Blastocystis[tiab] OR Sarcocystis[tiab] OR "Cyclospora cayetanensis"[tiab] OR tiab OR "Entamoeba histolytica"[tiab] OR "Balantidium coli"[tiab] OR Trichinellosis[tiab] OR Trichinosis[tiab] OR Trichinella[tiab] OR Toxoplasmosis[tiab] OR TORCH[tiab] OR Toxoplasma[tiab] OR Toxocarosis[tiab] OR Toxocariasis[tiab] OR Toxocariosis[tiab] OR "Larva migrans"[tiab] OR Toxocara[tiab] OR Taeniosis[tiab] OR Taeniasis[tiab] OR Tapeworm[tiab] OR Tapeworms[tiab] OR Taenia[tiab] OR "Foodborne trematodosis"[tiab] OR trematodosis[tiab] OR Trematodiasis[tiab] OR Fasciolosis[tiab] OR Fascioliosis[tiab] OR Fasciolasis[tiab] OR Fascioliasis[tiab] OR Distomatosis[tiab] OR Fasciolopsosis[tiab] OR Fasciolopsiosis[tiab] OR Opisthorchosis[tiab] OR Opisthorchiasis[tiab] OR Clonorchiosis[tiab] OR Clonorchiasis[tiab] OR Paragonimosis[tiab] OR Paragonimiasis[tiab] OR Metagonimus[tiab] OR Heterophyiasis[tiab] OR Fluke[tiab] OR Flukes[tiab] OR Trematode[tiab] OR Trematodes[tiab] OR Fasciola[tiab] OR Fasciolopsis[tiab] OR Opisthorchis[tiab] OR Clonorchis[tiab] OR Paragonimus[tiab] OR "Minute intestinal fluke"[tiab] OR "Minute intestinal flukes"[tiab] OR "Haplorchis pumilio"[tiab] OR "Metagonimus yokogawai"[tiab] OR "Heterophyes"[tiab] OR Diphyllobothriosis[tiab] OR Diphyllobothriasis[tiab] OR Bothriocephalosis[tiab] OR Bothriocephaliasis[tiab] OR Diphyllobothrium[tiab] OR Bothriocephalus[tiab] OR "Broad tapeworm"[tiab] OR "Broad tapeworms"[tiab] OR "Fish tapeworm"[tiab] OR "Fish tapeworms"[tiab] OR Cysticercosis[tiab] OR Neurocysticercosis[tiab] OR "Taenia solium"[tiab] OR "Cystic echinococcosis"[tiab] OR "Hydatid disease"[tiab] OR "Hydatid diseases"[tiab] OR Hydatidosis[tiab] OR "Echinococcus granulosus"[tiab] OR "Hydatid cyst"[tiab] OR "Hydatid cysts"[tiab] OR "Alveolar echinococcosis"[tiab] OR "Alveolar hydatidosis"[tiab] OR "Echinococcus multilocularis"[tiab]) AND (nomad*[tiab] OR nomadic[tiab] OR pastoralis*[tiab] OR herder*[tiab] OR "semi-nomadic"[tiab] OR pastoral[tiab] OR nomadism[tiab] OR transhumance[tiab] OR transhumant[tiab] OR agropastoralist*[tiab] OR "agro-pastoralist"[tiab] OR "agro-pastoralists"[tiab]) | 163 |
| Agricola | ab((“Alveolar echinococcosis” OR “Alveolar hydatidosis” OR “Echinococcus multilocularis*”*  OR Angiostrongylosis OR Angiostrongyliasis OR “Angiostrongylus cantonensis” OR “Anisakidae infections” OR “Anisakis pseudoterranova” OR Capillariosis OR Capillariasis OR Capillaria OR “Cystic echinococcosis” OR “Hydatid disease” OR “Hydatid diseases” OR Hydatidosis OR “Echinococcus granulosus” OR “Hydatid cyst” OR “Hydatid cysts” OR Cysticercosis OR Neurocysticercosis OR “Taenia solium” OR Diphyllobothriosis OR Diphyllobothriasis OR Bothriocephalosis OR Bothriocephaliasis OR Diphyllobothrium OR Bothriocephalus OR “Broad tapeworm” OR “Broad tapeworms” OR “Fish tapeworm” OR “Fish tapeworms” OR “Foodborne trematodosis” OR Trematodiasis OR Fasciolosis OR Fascioliosis OR Fasciolasis OR Fascioliasis OR Distomatosis OR Fasciolopsosis OR Fasciolopsiosis OR Opisthorchosis OR Opisthorchiasis OR Clonorchiosis OR Clonorchiasis OR Paragonimosis OR Paragonimiasis OR Metagonimus OR Heterophyiasis OR Fluke OR Flukes OR Trematode OR Trematodes OR Fasciola OR Fasciolopsis OR Opisthorchis OR Clonorchis OR Paragonimus OR “Minute intestinal fluke” OR “Minute intestinal flukes” OR “Haplorchis pumilio” OR “Metagonimus yokogawai” OR “Heterophyes” OR Gnathostomosis OR Gnathostomiasis OR Gnathostoma OR Sparganosis OR Spirometrosis OR Spirometra OR Sparganum OR Taeniosis OR Taeniasis OR Tapeworm OR Tapeworms OR Taenia OR Toxocarosis OR Toxocariasis OR Toxocariosis OR “Larva migrans” OR Toxocara OR Toxoplasmosis OR TORCH OR Toxoplasma OR Trichinellosis OR Trichinosis OR Trichinella OR “Zoonotic intestinal protozoal infection” OR “Zoonotic intestinal protozoal infections” OR Protozoosis OR Protozoasis OR Giardiosis OR Giardiasis OR Cryptosporidiosis OR Blastocystosis OR Sarcocystosis OR Cyclosporiasis OR Cyclospora OR Amoebiasis OR “Amoebic dysentery” OR Entamoeba OR Balantidosis OR Protozoa OR Giardia OR Cryptosporidium OR Blastocystis OR Sarcocystis OR “Cyclospora cayetanensis” OR “Entamoeba histolytica” OR “Balantidium coli” OR “Zoonotic schistosomosis” OR Schistosomiasis OR Bilharziosis OR “Snail fever” OR “Swimmer itch” OR “Swimmers itch” OR Schistosoma OR Bilharzia OR “Zoonotic trypanosomosis” OR Trypanosomiasis OR Chagas OR “Trypanosoma cruzi” OR “Zoonotic intestinal helminth infection” OR “Zoonotic intestinal helminth infections” OR Helminthosis OR Helminthiasis OR Ascarosis OR Ascariasis OR Ancylostomosis OR Ancylostomiasis OR Trichuriosis OR Trichuriasis OR Strongyloidosis OR Strongyloidiasis OR Helminth* OR Ascaris OR Ancylostoma OR Hookworm* OR Trichuris OR Strongyloides OR Alaria OR “rat lungworm” OR “rat lungworms” OR “Echinostoma” OR “Lagochilascaris minor” OR “Zoonotic microspore” OR “Zoonotic microspores” OR “Microsporidia” OR “Enterocytozooan bieneusi” OR “Encephalitozoon cuniculi” OR “Encephalitozoon intestinalis” OR “Encephalitozoon hellem” OR “Pleistophora-like organism” OR “Pleistophora-like organisms” OR “Zoonotic pentasome” OR “Zoonotic pentasomes” OR Pentastomiasis OR Linguatulosis OR “Armillifer armillatus” OR “Armillifer moniliformis” OR “Linguatula serrate” OR “Zoonotic enteric pathogen” OR “Zoonotic enteric pathogens” OR “Zoonotic enteric parasite” OR “Zoonotic enteric parasites” OR “Zoonotic parasite” OR “Zoonotic parasites” OR “Animal-to-Human parasite” OR“Animal-to-Human parasites” OR “Foodborne parasite” OR “Foodborne parasites” OR “Waterborne parasite” OR “Waterborne parasites” OR “Water-related parasite” OR “Water-related parasites” OR “Enteric parasite” OR “Enteric parasites” OR parasitism) AND (nomad* OR nomadic OR pastoralis* OR herder* OR “semi-nomadic” OR pastoral OR nomadism OR transhumance OR transhumant OR agropastoralist* OR “agro-pastoralist” OR “agro-pastoralists”)) | 31 |
| Web of Science: Core Collection | ((“Alveolar echinococcosis” OR “Alveolar hydatidosis” OR “Echinococcus multilocularis*”*  OR Angiostrongylosis OR Angiostrongyliasis OR “Angiostrongylus cantonensis” OR “Anisakidae infections” OR “Anisakis pseudoterranova” OR Capillariosis OR Capillariasis OR Capillaria OR “Cystic echinococcosis” OR “Hydatid disease” OR “Hydatid diseases” OR Hydatidosis OR “Echinococcus granulosus” OR “Hydatid cyst” OR “Hydatid cysts” OR Cysticercosis OR Neurocysticercosis OR “Taenia solium” OR Diphyllobothriosis OR Diphyllobothriasis OR Bothriocephalosis OR Bothriocephaliasis OR Diphyllobothrium OR Bothriocephalus OR “Broad tapeworm” OR “Broad tapeworms” OR “Fish tapeworm” OR “Fish tapeworms” OR “Foodborne trematodosis” OR Trematodiasis OR Fasciolosis OR Fascioliosis OR Fasciolasis OR Fascioliasis OR Distomatosis OR Fasciolopsosis OR Fasciolopsiosis OR Opisthorchosis OR Opisthorchiasis OR Clonorchiosis OR Clonorchiasis OR Paragonimosis OR Paragonimiasis OR Metagonimus OR Heterophyiasis OR Fluke OR Flukes OR Trematode OR Trematodes OR Fasciola OR Fasciolopsis OR Opisthorchis OR Clonorchis OR Paragonimus OR “Minute intestinal fluke” OR “Minute intestinal flukes” OR “Haplorchis pumilio” OR “Metagonimus yokogawai” OR “Heterophyes” OR Gnathostomosis OR Gnathostomiasis OR Gnathostoma OR Sparganosis OR Spirometrosis OR Spirometra OR Sparganum OR Taeniosis OR Taeniasis OR Tapeworm OR Tapeworms OR Taenia OR Toxocarosis OR Toxocariasis OR Toxocariosis OR “Larva migrans” OR Toxocara OR Toxoplasmosis OR TORCH OR Toxoplasma OR Trichinellosis OR Trichinosis OR Trichinella OR “Zoonotic intestinal protozoal infection” OR “Zoonotic intestinal protozoal infections” OR Protozoosis OR Protozoasis OR Giardiosis OR Giardiasis OR Cryptosporidiosis OR Blastocystosis OR Sarcocystosis OR Cyclosporiasis OR Cyclospora OR Amoebiasis OR “Amoebic dysentery” OR Entamoeba OR Balantidosis OR Protozoa OR Giardia OR Cryptosporidium OR Blastocystis OR Sarcocystis OR “Cyclospora cayetanensis” OR “Entamoeba histolytica” OR “Balantidium coli” OR “Zoonotic schistosomosis” OR Schistosomiasis OR Bilharziosis OR “Snail fever” OR “Swimmer itch” OR “Swimmers itch” OR Schistosoma OR Bilharzia OR “Zoonotic trypanosomosis” OR Trypanosomiasis OR Chagas OR “Trypanosoma cruzi” OR “Zoonotic intestinal helminth infection” OR “Zoonotic intestinal helminth infections” OR Helminthosis OR Helminthiasis OR Ascarosis OR Ascariasis OR Ancylostomosis OR Ancylostomiasis OR Trichuriosis OR Trichuriasis OR Strongyloidosis OR Strongyloidiasis OR Helminth* OR Ascaris OR Ancylostoma OR Hookworm* OR Trichuris OR Strongyloides OR Alaria OR “rat lungworm” OR “rat lungworms” OR “Echinostoma” OR “Lagochilascaris minor” OR “Zoonotic microspore” OR “Zoonotic microspores” OR “Microsporidia” OR “Enterocytozooan bieneusi” OR “Encephalitozoon cuniculi” OR “Encephalitozoon intestinalis” OR “Encephalitozoon hellem” OR “Pleistophora-like organism” OR “Pleistophora-like organisms” OR “Zoonotic pentasome” OR “Zoonotic pentasomes” OR Pentastomiasis OR Linguatulosis OR “Armillifer armillatus” OR “Armillifer moniliformis” OR “Linguatula serrate” OR “Zoonotic enteric pathogen” OR “Zoonotic enteric pathogens” OR “Zoonotic enteric parasite” OR “Zoonotic enteric parasites” OR “Zoonotic parasite” OR “Zoonotic parasites” OR “Animal-to-Human parasite” OR “Animal-to-Human parasites” OR “Foodborne parasite” OR “Foodborne parasites” OR “Waterborne parasite” OR “Waterborne parasites” OR “Water-related parasite” OR “Water-related parasites” OR “Enteric parasite” OR “Enteric parasites” OR parasitism) AND (nomad* OR nomadic OR pastoralis* OR herder* OR “semi-nomadic” OR pastoral OR nomadism OR transhumance OR transhumant OR agropastoralist* OR “agro-pastoralist” OR “agro-pastoralists”)) | 200 |
| Web of Science: Zoological Record | ((“Alveolar echinococcosis” OR “Alveolar hydatidosis” OR “Echinococcus multilocularis*”*  OR Angiostrongylosis OR Angiostrongyliasis OR “Angiostrongylus cantonensis” OR “Anisakidae infections” OR “Anisakis pseudoterranova” OR Capillariosis OR Capillariasis OR Capillaria OR “Cystic echinococcosis” OR “Hydatid disease” OR “Hydatid diseases” OR Hydatidosis OR “Echinococcus granulosus” OR “Hydatid cyst” OR “Hydatid cysts” OR Cysticercosis OR Neurocysticercosis OR “Taenia solium” OR Diphyllobothriosis OR Diphyllobothriasis OR Bothriocephalosis OR Bothriocephaliasis OR Diphyllobothrium OR Bothriocephalus OR “Broad tapeworm” OR “Broad tapeworms” OR “Fish tapeworm” OR “Fish tapeworms” OR “Foodborne trematodosis” OR Trematodiasis OR Fasciolosis OR Fascioliosis OR Fasciolasis OR Fascioliasis OR Distomatosis OR Fasciolopsosis OR Fasciolopsiosis OR Opisthorchosis OR Opisthorchiasis OR Clonorchiosis OR Clonorchiasis OR Paragonimosis OR Paragonimiasis OR Metagonimus OR Heterophyiasis OR Fluke OR Flukes OR Trematode OR Trematodes OR Fasciola OR Fasciolopsis OR Opisthorchis OR Clonorchis OR Paragonimus OR “Minute intestinal fluke” OR “Minute intestinal flukes” OR “Haplorchis pumilio” OR “Metagonimus yokogawai” OR “Heterophyes” OR Gnathostomosis OR Gnathostomiasis OR Gnathostoma OR Sparganosis OR Spirometrosis OR Spirometra OR Sparganum OR Taeniosis OR Taeniasis OR Tapeworm OR Tapeworms OR Taenia OR Toxocarosis OR Toxocariasis OR Toxocariosis OR “Larva migrans” OR Toxocara OR Toxoplasmosis OR TORCH OR Toxoplasma OR Trichinellosis OR Trichinosis OR Trichinella OR “Zoonotic intestinal protozoal infection” OR “Zoonotic intestinal protozoal infections” OR Protozoosis OR Protozoasis OR Giardiosis OR Giardiasis OR Cryptosporidiosis OR Blastocystosis OR Sarcocystosis OR Cyclosporiasis OR Cyclospora OR Amoebiasis OR “Amoebic dysentery” OR Entamoeba OR Balantidosis OR Protozoa OR Giardia OR Cryptosporidium OR Blastocystis OR Sarcocystis OR “Cyclospora cayetanensis” OR “Entamoeba histolytica” OR “Balantidium coli” OR “Zoonotic schistosomosis” OR Schistosomiasis OR Bilharziosis OR “Snail fever” OR “Swimmer itch” OR “Swimmers itch” OR Schistosoma OR Bilharzia OR “Zoonotic trypanosomosis” OR Trypanosomiasis OR Chagas OR “Trypanosoma cruzi” OR “Zoonotic intestinal helminth infection” OR “Zoonotic intestinal helminth infections” OR Helminthosis OR Helminthiasis OR Ascarosis OR Ascariasis OR Ancylostomosis OR Ancylostomiasis OR Trichuriosis OR Trichuriasis OR Strongyloidosis OR Strongyloidiasis OR Helminth* OR Ascaris OR Ancylostoma OR Hookworm* OR Trichuris OR Strongyloides OR Alaria OR “rat lungworm” OR “rat lungworms” OR “Echinostoma” OR “Lagochilascaris minor” OR “Zoonotic microspore” OR “Zoonotic microspores” OR “Microsporidia” OR “Enterocytozooan bieneusi” OR “Encephalitozoon cuniculi” OR “Encephalitozoon intestinalis” OR “Encephalitozoon hellem” OR “Pleistophora-like organism” OR “Pleistophora-like organisms” OR “Zoonotic pentasome” OR “Zoonotic pentasomes” OR Pentastomiasis OR Linguatulosis OR “Armillifer armillatus” OR “Armillifer moniliformis” OR “Linguatula serrate” OR “Zoonotic enteric pathogen” OR “Zoonotic enteric pathogens” OR “Zoonotic enteric parasite” OR “Zoonotic enteric parasites” OR “Zoonotic parasite” OR “Zoonotic parasites” OR “Animal-to-Human parasite” OR “Animal-to-Human parasites” OR “Foodborne parasite” OR “Foodborne parasites” OR “Waterborne parasite” OR “Waterborne parasites” OR “Water-related parasite” OR “Water-related parasites” OR “Enteric parasite” OR “Enteric parasites” OR parasitism) AND (nomad* OR nomadic OR pastoralis* OR herder* OR “semi-nomadic” OR pastoral OR nomadism OR transhumance OR transhumant OR agropastoralist* OR “agro-pastoralist” OR “agro-pastoralists”)) | 105 |
| Web of Science: CABI | ((“Alveolar echinococcosis” OR “Alveolar hydatidosis” OR “Echinococcus multilocularis*”*  OR Angiostrongylosis OR Angiostrongyliasis OR “Angiostrongylus cantonensis” OR “Anisakidae infections” OR “Anisakis pseudoterranova” OR Capillariosis OR Capillariasis OR Capillaria OR “Cystic echinococcosis” OR “Hydatid disease” OR “Hydatid diseases” OR Hydatidosis OR “Echinococcus granulosus” OR “Hydatid cyst” OR “Hydatid cysts” OR Cysticercosis OR Neurocysticercosis OR “Taenia solium” OR Diphyllobothriosis OR Diphyllobothriasis OR Bothriocephalosis OR Bothriocephaliasis OR Diphyllobothrium OR Bothriocephalus OR “Broad tapeworm” OR “Broad tapeworms” OR “Fish tapeworm” OR “Fish tapeworms” OR “Foodborne trematodosis” OR Trematodiasis OR Fasciolosis OR Fascioliosis OR Fasciolasis OR Fascioliasis OR Distomatosis OR Fasciolopsosis OR Fasciolopsiosis OR Opisthorchosis OR Opisthorchiasis OR Clonorchiosis OR Clonorchiasis OR Paragonimosis OR Paragonimiasis OR Metagonimus OR Heterophyiasis OR Fluke OR Flukes OR Trematode OR Trematodes OR Fasciola OR Fasciolopsis OR Opisthorchis OR Clonorchis OR Paragonimus OR “Minute intestinal fluke” OR “Minute intestinal flukes” OR “Haplorchis pumilio” OR “Metagonimus yokogawai” OR “Heterophyes” OR Gnathostomosis OR Gnathostomiasis OR Gnathostoma OR Sparganosis OR Spirometrosis OR Spirometra OR Sparganum OR Taeniosis OR Taeniasis OR Tapeworm OR Tapeworms OR Taenia OR Toxocarosis OR Toxocariasis OR Toxocariosis OR “Larva migrans” OR Toxocara OR Toxoplasmosis OR TORCH OR Toxoplasma OR Trichinellosis OR Trichinosis OR Trichinella OR “Zoonotic intestinal protozoal infection” OR “Zoonotic intestinal protozoal infections” OR Protozoosis OR Protozoasis OR Giardiosis OR Giardiasis OR Cryptosporidiosis OR Blastocystosis OR Sarcocystosis OR Cyclosporiasis OR Cyclospora OR Amoebiasis OR “Amoebic dysentery” OR Entamoeba OR Balantidosis OR Protozoa OR Giardia OR Cryptosporidium OR Blastocystis OR Sarcocystis OR “Cyclospora cayetanensis” OR “Entamoeba histolytica” OR “Balantidium coli” OR “Zoonotic schistosomosis” OR Schistosomiasis OR Bilharziosis OR “Snail fever” OR “Swimmer itch” OR “Swimmers itch” OR Schistosoma OR Bilharzia OR “Zoonotic trypanosomosis” OR Trypanosomiasis OR Chagas OR “Trypanosoma cruzi” OR “Zoonotic intestinal helminth infection” OR “Zoonotic intestinal helminth infections” OR Helminthosis OR Helminthiasis OR Ascarosis OR Ascariasis OR Ancylostomosis OR Ancylostomiasis OR Trichuriosis OR Trichuriasis OR Strongyloidosis OR Strongyloidiasis OR Helminth* OR Ascaris OR Ancylostoma OR Hookworm* OR Trichuris OR Strongyloides OR Alaria OR “rat lungworm” OR “rat lungworms” OR “Echinostoma” OR “Lagochilascaris minor” OR “Zoonotic microspore” OR “Zoonotic microspores” OR “Microsporidia” OR “Enterocytozooan bieneusi” OR “Encephalitozoon cuniculi” OR “Encephalitozoon intestinalis” OR “Encephalitozoon hellem” OR “Pleistophora-like organism” OR “Pleistophora-like organisms” OR “Zoonotic pentasome” OR “Zoonotic pentasomes” OR Pentastomiasis OR Linguatulosis OR “Armillifer armillatus” OR “Armillifer moniliformis” OR “Linguatula serrate” OR “Zoonotic enteric pathogen” OR “Zoonotic enteric pathogens” OR “Zoonotic enteric parasite” OR “Zoonotic enteric parasites” OR “Zoonotic parasite” OR “Zoonotic parasites” OR “Animal-to-Human parasite” OR “Animal-to-Human parasites” OR “Foodborne parasite” OR “Foodborne parasites” OR “Waterborne parasite” OR “Waterborne parasites” OR “Water-related parasite” OR “Water-related parasites” OR “Enteric parasite” OR “Enteric parasites” OR parasitism) AND (nomad* OR nomadic OR pastoralis* OR herder* OR “semi-nomadic” OR pastoral OR nomadism OR transhumance OR transhumant OR agropastoralist* OR “agro-pastoralist” OR “agro-pastoralists”)) | 959 |
| BIOSIS | ((“Alveolar echinococcosis” OR “Alveolar hydatidosis” OR “Echinococcus multilocularis*”*  OR Angiostrongylosis OR Angiostrongyliasis OR “Angiostrongylus cantonensis” OR “Anisakidae infections” OR “Anisakis pseudoterranova” OR Capillariosis OR Capillariasis OR Capillaria OR “Cystic echinococcosis” OR “Hydatid disease” OR “Hydatid diseases” OR Hydatidosis OR “Echinococcus granulosus” OR “Hydatid cyst” OR “Hydatid cysts” OR Cysticercosis OR Neurocysticercosis OR “Taenia solium” OR Diphyllobothriosis OR Diphyllobothriasis OR Bothriocephalosis OR Bothriocephaliasis OR Diphyllobothrium OR Bothriocephalus OR “Broad tapeworm” OR “Broad tapeworms” OR “Fish tapeworm” OR “Fish tapeworms” OR “Foodborne trematodosis” OR Trematodiasis OR Fasciolosis OR Fascioliosis OR Fasciolasis OR Fascioliasis OR Distomatosis OR Fasciolopsosis OR Fasciolopsiosis OR Opisthorchosis OR Opisthorchiasis OR Clonorchiosis OR Clonorchiasis OR Paragonimosis OR Paragonimiasis OR Metagonimus OR Heterophyiasis OR Fluke OR Flukes OR Trematode OR Trematodes OR Fasciola OR Fasciolopsis OR Opisthorchis OR Clonorchis OR Paragonimus OR “Minute intestinal fluke” OR “Minute intestinal flukes” OR “Haplorchis pumilio” OR “Metagonimus yokogawai” OR “Heterophyes” OR Gnathostomosis OR Gnathostomiasis OR Gnathostoma OR Sparganosis OR Spirometrosis OR Spirometra OR Sparganum OR Taeniosis OR Taeniasis OR Tapeworm OR Tapeworms OR Taenia OR Toxocarosis OR Toxocariasis OR Toxocariosis OR “Larva migrans” OR Toxocara OR Toxoplasmosis OR TORCH OR Toxoplasma OR Trichinellosis OR Trichinosis OR Trichinella OR “Zoonotic intestinal protozoal infection” OR “Zoonotic intestinal protozoal infections” OR Protozoosis OR Protozoasis OR Giardiosis OR Giardiasis OR Cryptosporidiosis OR Blastocystosis OR Sarcocystosis OR Cyclosporiasis OR Cyclospora OR Amoebiasis OR “Amoebic dysentery” OR Entamoeba OR Balantidosis OR Protozoa OR Giardia OR Cryptosporidium OR Blastocystis OR Sarcocystis OR “Cyclospora cayetanensis” OR “Entamoeba histolytica” OR “Balantidium coli” OR “Zoonotic schistosomosis” OR Schistosomiasis OR Bilharziosis OR “Snail fever” OR “Swimmer itch” OR “Swimmers itch” OR Schistosoma OR Bilharzia OR “Zoonotic trypanosomosis” OR Trypanosomiasis OR Chagas OR “Trypanosoma cruzi” OR “Zoonotic intestinal helminth infection” OR “Zoonotic intestinal helminth infections” OR Helminthosis OR Helminthiasis OR Ascarosis OR Ascariasis OR Ancylostomosis OR Ancylostomiasis OR Trichuriosis OR Trichuriasis OR Strongyloidosis OR Strongyloidiasis OR Helminth* OR Ascaris OR Ancylostoma OR Hookworm* OR Trichuris OR Strongyloides OR Alaria OR “rat lungworm” OR “rat lungworms” OR “Echinostoma” OR “Lagochilascaris minor” OR “Zoonotic microspore” OR “Zoonotic microspores” OR “Microsporidia” OR “Enterocytozooan bieneusi” OR “Encephalitozoon cuniculi” OR “Encephalitozoon intestinalis” OR “Encephalitozoon hellem” OR “Pleistophora-like organism” OR “Pleistophora-like organisms” OR “Zoonotic pentasome” OR “Zoonotic pentasomes” OR Pentastomiasis OR Linguatulosis OR “Armillifer armillatus” OR “Armillifer moniliformis” OR “Linguatula serrate” OR “Zoonotic enteric pathogen” OR “Zoonotic enteric pathogens” OR “Zoonotic enteric parasite” OR “Zoonotic enteric parasites” OR “Zoonotic parasite” OR “Zoonotic parasites” OR “Animal-to-Human parasite” OR “Animal-to-Human parasites” OR “Foodborne parasite” OR “Foodborne parasites” OR “Waterborne parasite” OR “Waterborne parasites” OR “Water-related parasite” OR “Water-related parasites” OR “Enteric parasite” OR “Enteric parasites” OR parasitism) AND (nomad* OR nomadic OR pastoralis* OR herder* OR “semi-nomadic” OR pastoral OR nomadism OR transhumance OR transhumant OR agropastoralist* OR “agro-pastoralist” OR “agro-pastoralists”)) | 310 |
| Proquest: 12 databases including ProQuest Agricultural Science Collection‎, ProQuest Aquatic Science Collection‎, ProQuest Biological Science Collection‎, ProQuest Earth Science Collection‎, ProQuest Environmental Science Collection‎, COS Conference Papers Index‎, Health & Safety Science Abstracts‎, MEDLINE®‎, TOXLINE | ab((“Alveolar echinococcosis” OR “Alveolar hydatidosis” OR “*Echinococcus multilocularis”*  OR Angiostrongylosis OR Angiostrongyliasis OR “Angiostrongylus cantonensis” OR “Anisakidae infections” OR “Anisakis pseudoterranova” OR Capillariosis OR Capillariasis OR Capillaria OR “Cystic echinococcosis” OR “Hydatid disease” OR “Hydatid diseases” OR Hydatidosis OR “Echinococcus granulosus” OR “Hydatid cyst” OR “Hydatid cysts” OR Cysticercosis OR Neurocysticercosis OR “Taenia solium” OR Diphyllobothriosis OR Diphyllobothriasis OR Bothriocephalosis OR Bothriocephaliasis OR Diphyllobothrium OR Bothriocephalus OR “Broad tapeworm” OR “Broad tapeworms” OR “Fish tapeworm” OR “Fish tapeworms” OR “Foodborne trematodosis” OR Trematodiasis OR Fasciolosis OR Fascioliosis OR Fasciolasis OR Fascioliasis OR Distomatosis OR Fasciolopsosis OR Fasciolopsiosis OR Opisthorchosis OR Opisthorchiasis OR Clonorchiosis OR Clonorchiasis OR Paragonimosis OR Paragonimiasis OR Metagonimus OR Heterophyiasis OR Fluke OR Flukes OR Trematode OR Trematodes OR Fasciola OR Fasciolopsis OR Opisthorchis OR Clonorchis OR Paragonimus OR “Minute intestinal fluke” OR “Minute intestinal flukes” OR “Haplorchis pumilio” OR “Metagonimus yokogawai” OR “Heterophyes” OR Gnathostomosis OR Gnathostomiasis OR Gnathostoma OR Sparganosis OR Spirometrosis OR Spirometra OR Sparganum OR Taeniosis OR Taeniasis OR Tapeworm OR Tapeworms OR Taenia OR Toxocarosis OR Toxocariasis OR Toxocariosis OR “Larva migrans” OR Toxocara OR Toxoplasmosis OR TORCH OR Toxoplasma OR Trichinellosis OR Trichinosis OR Trichinella OR “Zoonotic intestinal protozoal infection” OR “Zoonotic intestinal protozoal infections” OR Protozoosis OR Protozoasis OR Giardiosis OR Giardiasis OR Cryptosporidiosis OR Blastocystosis OR Sarcocystosis OR Cyclosporiasis OR Cyclospora OR Amoebiasis OR “Amoebic dysentery” OR Entamoeba OR Balantidosis OR Protozoa OR Giardia OR Cryptosporidium OR Blastocystis OR Sarcocystis OR “Cyclospora cayetanensis” OR “Entamoeba histolytica” OR “Balantidium coli” OR “Zoonotic schistosomosis” OR Schistosomiasis OR Bilharziosis OR “Snail fever” OR “Swimmer itch” OR “Swimmers itch” OR Schistosoma OR Bilharzia OR “Zoonotic trypanosomosis” OR Trypanosomiasis OR Chagas OR “Trypanosoma cruzi” OR “Zoonotic intestinal helminth infection” OR “Zoonotic intestinal helminth infections” OR Helminthosis OR Helminthiasis OR Ascarosis OR Ascariasis OR Ancylostomosis OR Ancylostomiasis OR Trichuriosis OR Trichuriasis OR Strongyloidosis OR Strongyloidiasis OR Helminth* OR Ascaris OR Ancylostoma OR Hookworm* OR Trichuris OR Strongyloides OR Alaria OR “rat lungworm” OR “rat lungworms” OR “Echinostoma” OR “Lagochilascaris minor” OR “Zoonotic microspore” OR “Zoonotic microspores” OR “Microsporidia” OR “Enterocytozooan bieneusi” OR “Encephalitozoon cuniculi” OR “Encephalitozoon intestinalis” OR “Encephalitozoon hellem” OR “Pleistophora-like organism” OR “Pleistophora-like organisms” OR “Zoonotic pentasome” OR “Zoonotic pentasomes” OR Pentastomiasis OR Linguatulosis OR “Armillifer armillatus” OR “Armillifer moniliformis” OR “Linguatula serrate” OR “Zoonotic enteric pathogen” OR “Zoonotic enteric pathogens” OR “Zoonotic enteric parasite” OR “Zoonotic enteric parasites” OR “Zoonotic parasite” OR “Zoonotic parasites” OR “Animal-to-Human parasite” OR“Animal-to-Human parasites” OR “Foodborne parasite” OR “Foodborne parasites” OR “Waterborne parasite” OR “Waterborne parasites” OR “Water-related parasite” OR “Water-related parasites” OR “Enteric parasite” OR “Enteric parasites” OR parasitism) AND (nomad* OR nomadic OR pastoralis* OR herder* OR “semi-nomadic” OR pastoral OR nomadism OR transhumance OR transhumant OR agropastoralist* OR “agro-pastoralist” OR “agro-pastoralists”)) | 289 with 162 available for citation referencing |
| **Total Results:** | | 1,930 |
